# Editorial: Bringing together data- and knowledge-driven solutions for a better understanding and effective diagnostics of neurological disorders

**DOI:** 10.3389/fninf.2023.1229945

**Published:** 2023-07-20

**Authors:** Dmitrii Kaplun, Mikhail Bogachev, Pawan Kumar Singh, Ram Sarkar

**Affiliations:** ^1^Department of Automation and Control Processes, Saint Petersburg Electrotechnical University “LETI”, Saint Petersburg, Russia; ^2^Centre for Digital Telecommunication Technologies, Saint Petersburg Electrotechnical University “LETI”, Saint Petersburg, Russia; ^3^Department of Information Technology, Jadavpur University, Kolkata, West Bengal, India; ^4^Department of Computer Science and Engineering, Jadavpur University, Kolkata, West Bengal, India

**Keywords:** artificial intelligence, data-driven models, knowledge-driven models, neurological disorders, interpretable functional models

Modern artificial intelligence approaches have been largely inspired by biological neural networks, and thus there is no surprise that they found extensive applications in the understanding of neurological disorders. Nowadays highly efficient diagnostic methods are largely based on deep learning procedures which, due to their inherent complexity, could hardly be understood any longer, being increasingly represented by black-box-style solutions, that appears among key limitations preventing their further translation into clinical practice. Finding successful integrative solutions where modern artificial intelligence approaches are complemented by functional models with few parameters that could be directly interpreted by medical practitioners represent one of the key challenges. Potential approaches to the problem include, while are not limited to, combination of unsupervised data-driven with supervised knowledge-driven models, functional model with few directly interpretable parameters obtained using statistical analysis and/or dimensionality reduction methods, with applications ranging from retrospective analysis to experimental conditions and clinical applications.

The goal of this Research Topic is to address the above issues by bringing together analytical, computational, and statistical approaches, this way facilitating the development of both efficient and interpretable neurophysiological models, leading to a better understanding of neurological disorders. Thus, the overall aim is to improve early diagnostic and management control strategies, altogether contributing to the increased effectiveness of experimental and clinical data interpretation.

Particular contributions to this topic addressed the following aspects:

– Three papers in this Research Topic focus on the data-driven and knowledge-driven approaches to the diagnosis and modeling of neurodegenerative disorders.

In their paper entitled “*Interpretable evaluation for the Brunnstrom recovery stage of the lower limb based on wearable sensors*,” Chen et al. propose an interpretable BRS-L evaluation method based on wearable sensors. The authors collected lower limb motion data and plantar pressure data of 20 hemiplegic patients and 10 healthy individuals using seven Inertial Measurement Units and two plantar pressure insoles. Then the authors extracted gait features from the motion data and pressure data, and using feature selection based on feature importance, improved the interpretability of the results for several evaluated machine-learning methods, leading to a feasible solution for precise rehabilitation.

The paper entitled “*A hybrid unsupervised and supervised learning approach for postictal generalized EEG suppression detection*” by Li et al. focuses on a hybrid approach that combines the benefits of unsupervised and supervised learning for PGES detection using multi-channel EEG recordings. The authors introduce a new learning strategy for training a set of random forest (RF) models based on clustering results to improve PGES detection performance, leading to the enhancement of accuracy beyond the performance levels achieved by concurrent approaches.

Kustubayeva et al. in their contribution entitled “*Functional MRI study of feedback-based reinforcement learning in depression*” investigated whether depressive symptoms could be associated with abnormalities in the learning-related brain activity, as measured by the functional magnetic resonance imaging (fMRI), and whether melancholic and atypical features could somehow reflect possible alterations in the brain activity. The authors examined event-related brain activation during feedback-based learning task, leading to an observation that MDD patients exhibited reduced activation in visual cortex but increased activation in cingulate and insular regions compared to healthy participants. In addition, it is been revealed that the levels of activation in striatal, thalamic, and precuneus regions were negatively correlated with atypical characteristics, suggesting that the MDD affects the neural circuitry underlying associative learning, and these effects may depend upon subtype features of MDD.

Another focus of this Research Topic represents modeling concepts and their applications from retrospective analysis to experimental animal models and clinical applications, represented by a sole contribution entitled “*Video-based marker-free tracking and multi-scale analysis of mouse locomotor activity and behavioral aspects in an open field arena: a perspective approach to the quantification of complex gait disturbances associated with Alzheimer's disease*” by Bogachev et al.. This work presents a novel marker-free instrumental approach to the analysis of gait disturbances in animal models based on the analysis of video recordings obtained with a camera placed underneath an open field arena with transparent floor. The extraction of motion trajectories is based on a recently proposed machine learning based algorithm capable of online tracking of individual animal body parts, such as the snout, the paws and the tail. Further analysis of the trajectories is performed by original computerized methodology that relies upon a generalized scalable model based on fractional Brownian motion with parameters identified by detrended partial cross-correlation analysis. A universal animal movement model characterized by fluctuation functions exhibiting two asymptotic scaling regimes separated by a single crossover is proposed. Using alignment of trajectories, it has been revealed that gait disturbances are explicitly reflected in the delays of the cross-correlation maxima, that in turn are proportional to the stride times, and related to the stride length taking into account the average speed of the animal, with statistically significant discrepancies observed in the Alzheimer's disease mouse model compared to the control group.

The final focus on AI-based integrative solutions including practically oriented support systems for both experimental research and clinical diagnostics of neurological disorders is represented by the paper “*Neural network system for analyzing statistical factors of patients for predicting the survival of dental implants*” by Lyakhov et al., where an AI system for analyzing various patient statistics to predict the success of single implant survival is proposed. The key novelty of the proposed solution lies in the developed optimal NN architecture designed to recognize the collected and digitized database of various patient factors based on the description of the case histories. The proposed solution is characterized by high accuracy close to 95%, indicating its capability of predicting the outcome of a dental implant operation, and thus could be potentially useful for the improvement of decision-making in clinical settings.

In general, this Research Topic made it possible to look at the problems of diagnosing neurodegenerative diseases, rehabilitating patients and developing expert diagnostic systems based on heterogeneous data from different points of view: AI-based, statistics-based, sensors-based. New methods and approaches have been proposed for the Research Topic, analysis and interpretation of diagnostic and rehabilitation data.

## Author contributions

All authors listed have made a substantial, direct, and intellectual contribution to the work and approved it for publication.

